# Supplementary figures and images for: Y-Box Binding Protein 1 Interacts with Dengue Virus Nucleocapsid and Mediates Viral Assembly
Source: mBio. 2022 Feb 22;13(1):e00196-22. doi: 10.1128/mbio.00196-22 (PMC8903895; doi:10.1128/mbio.00196-22)

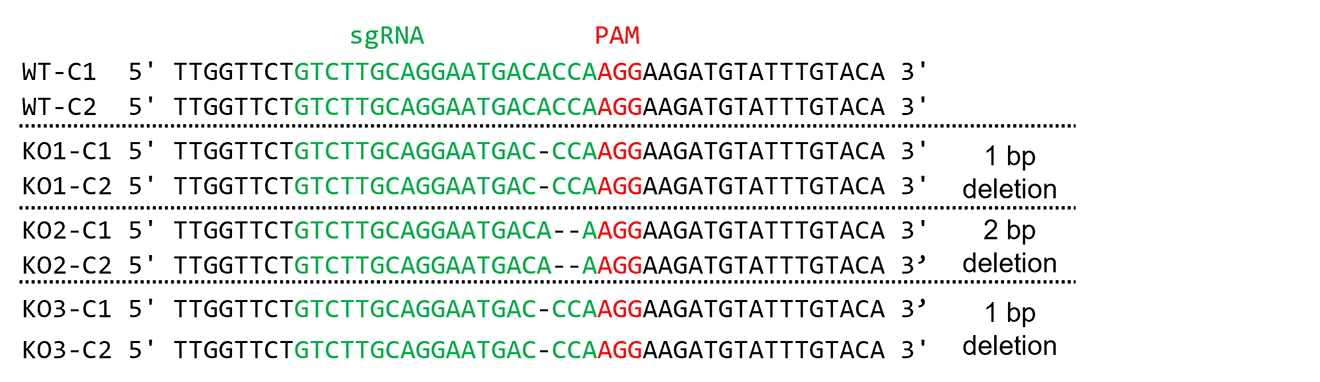

Supplement: FIG S1 [file mbio.00196-22-sf001.tif]

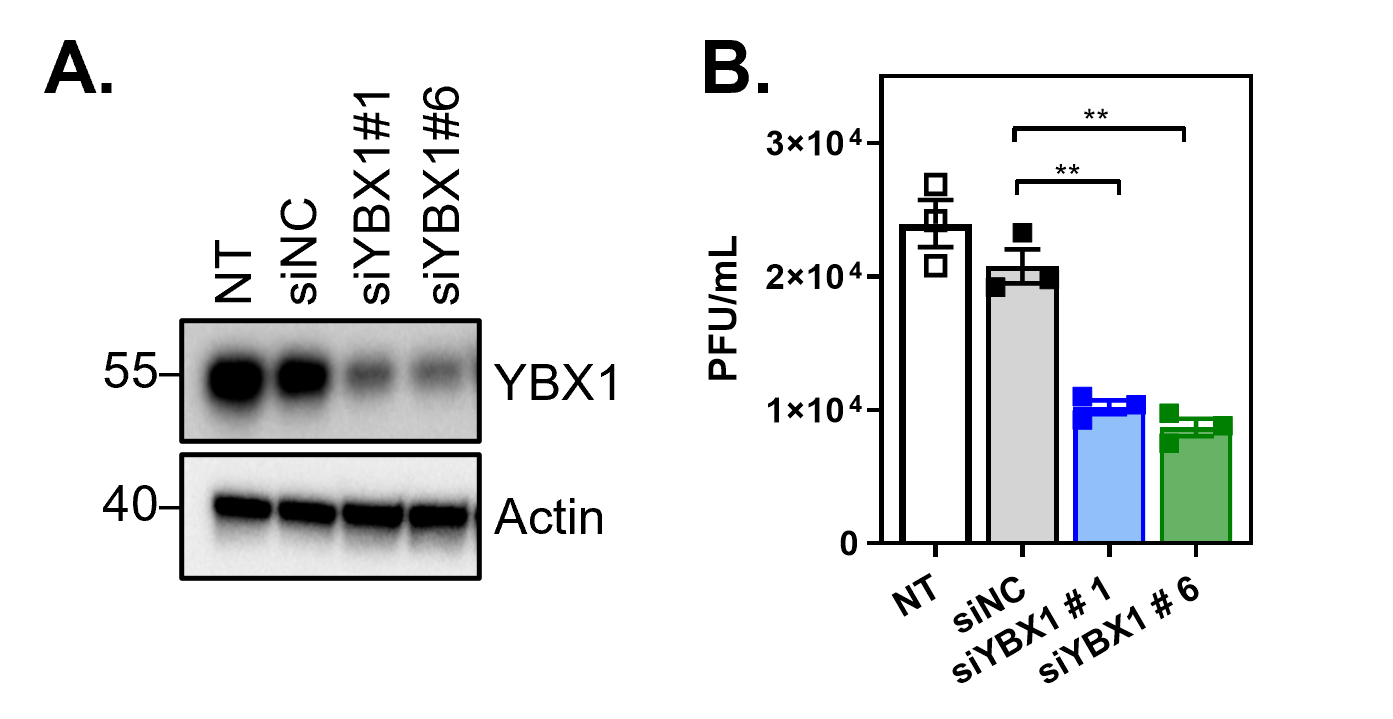

Supplement: FIG S2 [file mbio.00196-22-sf002.tif]
