# Supplementary material for: Y-Box Binding Protein 1 Interacts with Dengue Virus Nucleocapsid and Mediates Viral Assembly
Source: mBio. 2022 Feb 22;13(1):e00196-22. doi: 10.1128/mbio.00196-22 (PMC8903895; doi:10.1128/mbio.00196-22)
Supplement: TABLE S1 [file mbio.00196-22-st001.pdf]

**Supplemental material.**

**Supplementary table S1**. List of antibodies used.

| Antibody | Provider | Application | Dilution / quantity |
|---|---|---|---|
| YBX1 | Thermo Fisher Scientific | WB, IF | 1:1000, 1:500 |
| YBX1 | Abcam | RNA-IP | 5 µg |
| Actin | Thermo Fisher Scientific | WB | 1:2000 |
| dsRNA (J2) | Scicons | IF, PLA | 1:500; 1:4000 |
| Envelope | Invitrogen | WB, IF, PLA | 1:2000, 1:1000, 1:4000 |
| Capsid | Thermo Fisher Scientific | WB | 1:1000 |
| Capsid | Abcam | RNA-IP | 5 µg |
| prM | Genetex | WB, PLA | 1:1000, 1:4000 |
| Rabbit IgG | Abcam | RNA-IP | 5 µg |
| Calnexin | BD Biosciences | WB | 1:1000 |
| NS1 | Abcam | WB | 1:1000 |
| Hsp90 | Invitrogen | WB | 1:1000 |
| CD63 | Santa Cruz | WB | 1:500 |

*WB: western blot; RNA-IP: RNA immunoprecipitation; IF: immunofluorescence; PLA: proximity ligation assay.*
